# Rice Canopy Disease and Pest Identification Based on Improved YOLOv5 and UAV Images

**DOI:** 10.3390/s25134072

**Published:** 2025-06-30

**Authors:** Gaoyuan Zhao, Yubin Lan, Yali Zhang, Jizhong Deng

**Affiliations:** 1 College of Mechanical and Electrical Engineering, Lingnan Normal University, Zhanjiang 524048, China; 20211168015@stu.scau.edu.cn; 2 College of Engineering, South China Agricultural University, Guangzhou 510642, China; ylan@scau.edu.cn (Y.L.); ylzhang@scau.edu.cn (Y.Z.); 3 School of Artificial Intelligence, Zhujiang College, South China Agricultural University, Guangzhou 510980, China

**Keywords:** rice, canopy diseases and pests, UAV images, YOLOv5

## Abstract

Traditional monitoring methods rely on manual field surveys, which are subjective, inefficient, and unable to meet the demand for large-scale, rapid monitoring. By using unmanned aerial vehicles (UAVs) to capture high-resolution images of rice canopy diseases and pests, combined with deep learning (DL) techniques, accurate and timely identification of diseases and pests can be achieved. We propose a method for identifying rice canopy diseases and pests using an improved YOLOv5 model (YOLOv5_DWMix). By incorporating deep separable convolutions, the MixConv module, attention mechanisms, and optimized loss functions into the YOLOv5 backbone, the model’s speed, feature extraction capability, and robustness are significantly enhanced. Additionally, to tackle the challenges posed by complex field environments and small datasets, image augmentation is employed to train the YOLOv5_DWMix model for the recognition of four common rice canopy diseases and pests. Results show that the improved YOLOv5 model achieves 95.6% average precision in detecting these diseases and pests, a 4.8% improvement over the original YOLOv5 model. The YOLOv5_DWMix model is effective and advanced in identifying rice diseases and pests, offering a solid foundation for large-scale, regional monitoring.

## 1. Introduction

With continuous changes in the climate and rapid urban expansion, the problem of crop diseases and pests is escalating, and food security has become an urgent issue facing the world today. As one of the important staple foods in China, rice production is crucial for global food security. The serious constraint of diseases and pests hinders the development of rice in China. Traditional monitoring of rice diseases and pests mainly relies on manual field surveys, which are subjective, cumbersome, and time-consuming and cannot meet the practical demand for large-scale and rapid monitoring of diseases and pests [1,2]. Deep learning, with strong learning ability, adaptability, and portability, has been widely applied in crop disease and pest identification and classification. By preprocessing, feature extraction, and classifying disease and pest images, timely and accurate understanding of the types and occurrence areas of diseases and pests can help farmers take targeted control measures quickly, avoiding blind, erroneous, and excessive pesticide use, which is environmentally friendly and important for improving the quality of agricultural products [3,4,5,6]. Wang et al. [7] proposed an attention-based deep separable network model for classifying and detecting rice pests with an accuracy of 94.65%. Burhan et al. [8] conducted a comparative study on the classification performance of preprocessed rice datasets by removing backgrounds and shadows using five different models, and the results showed that ResNet101 V2 had the best performance, with an accuracy of 86.8%. Hu et al. [9] proposed an advanced YOLO-GBS model for the accurate detection of rice pests, with an average precision of 79.8% on a self-made dataset, which is 5.4% higher than the original YOLOv. Li et al. [10] developed a deep learning-based video detection architecture for identifying 16 types of diseases and pests affecting rice, such as rice blast, rice brown spot, and rice stem borers. Their custom backbone network outperformed other models like VGG50, ResNet-101, ResNet-3, and YOLOv25, especially in detecting slightly blurred images, demonstrating its potential for wider application in crop disease and pest detection. Most of the above research focuses on classifying or identifying diseases and pests on crop leaves, rather than meeting the need for rapid and real-time monitoring of diseases and pests in the canopy layer of rice on a large scale. Unmanned aerial vehicles (UAVs) equipped with high-resolution cameras can quickly and massively detect crop diseases and pests. However, research on rice disease and pest detection using drones is limited and mostly focuses on detecting a single disease or pest. Wang et al. [11] used a multi-rotor drone to collect visible light images for extracting features of white panicle images related to rice diseases and achieved an identification accuracy of 93.62% using the Adaboost algorithm. Wei et al. [12] used low-altitude drone remote sensing images and the YOLOv4 object detection deep neural network method to detect the severity of rice sheath blight, generating a rice density prescription map. Yao et al. [13] collected images of rice leaf rollers and rice stem borers at the plot scale during different growth stages and with different varieties using a high-resolution camera. They proposed an improved RetinaNet object detection model to support the intelligent and rapid monitoring of the two common pests in rice.

Due to the distant image acquisition distance of UAVs, the size of lesions is much smaller than those captured by handheld cameras. Based on this, this paper takes the four common diseases and pests in rice as research objects and proposes an improved YOLOv5-based rice canopy disease and pest identification model (YOLOv5_DWMix) to achieve effective identification of high-resolution rice canopy disease and pest images captured by UAVs. Building upon the YOLOv5 backbone network, deep separable convolutions are introduced to reduce the model’s parameters and computational load, thereby improving the model’s processing speed. MixConv is introduced to automatically select convolution kernel sizes suitable for different feature dimensions, enhancing the model’s capability to extract information from different lesion features and effectively improving the accuracy of rice canopy disease and pest identification. Other improvements include updating YOLOv5’s bounding box (B-Box) information using the k-means clustering algorithm, introducing the Convolutional Block Attention Module (CBAM) to adaptively weight feature maps, enhancing the extraction of effective feature information, and improving the quality of feature representation. In addition, the ComputeLossOTA loss function addresses matching issues through an optimal transport algorithm, ensuring each real target is matched with a predicted target, and better-handling variations in target shapes and sizes, as well as partial occlusions and incomplete targets. And LeakyReLU (0.1) helps alleviate gradient disappearance while reducing neuron death issues. Finally, considering the complexity of actual field environments and the tendency of small datasets to lead to overfitting, the original dataset undergoes image enhancement processes to train the YOLOv5_DWMix model to recognize the four common types of rice canopy diseases and pests.

The remainder of this paper is organized as follows. Section 2 describes the materials and methods used, including image collection of rice diseases and pests, as well as the construction of the dataset. Section 3 presents the models and training process, beginning with the YOLOv5 algorithm, followed by the improvements made to YOLOv5. This section also provides a detailed discussion of the model training and evaluation metrics, including the training platform, parameters, and evaluation metrics. Section 4 presents the results and analysis, comparing the performance of classic models, various attention mechanisms, and ablation experiments, as well as the identification results of rice diseases and pests using different models. Finally, Section 5 discusses the findings of the study, and Section 6 summarizes the research.

## 2. Materials and Methods

### 2.1. Image Collection of Rice Diseases and Pests

**Figure 1:**
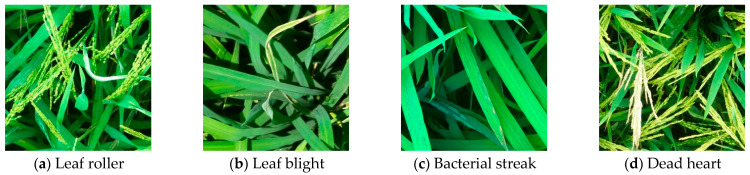
Rice canopy diseases and pests.

Common diseases and pests in the rice canopy include rice leaf roller (RLR), rice bacterial leaf blight (BLB), bacterial leaf streak (BLS), and dead heart. Using UAVs, 1000 images of rice canopy affected by diseases and pests were captured at flight heights ranging from 5 to 10 m, during the period from September 2021 to September 2022, under clear weather conditions with wind speeds below 3 m/s. The image collection took place in rice fields located in Guangdong Province, including areas in Zengcheng (Guangzhou) and Xinhui (Jiangmen). To ensure independent distribution of the dataset, the images underwent preprocessing such as cropping and selection, resulting in 600 annotated images for creating the rice canopy disease and pest dataset. Some image samples are shown in Figure 1. From Figure 1, it can be observed that the leaf lesions caused by RLR appear white due to feeding on the epidermis and mesophyll of the leaves, affecting rice photosynthesis and leading to yield reduction. Lesions affected by rice BLB mainly distribute at the leaf tips, caused by the pathogenic bacteria of rice BLB. The initial symptoms start with dark green, water-soaked short streaks at the leaf tip or margin, quickly turning dark brown and then forming light yellow-white lesions around the streaks, which continue to expand along the leaf margin or midrib, turning yellow-brown and eventually white. Symptoms of BLS initially manifest as small water-soaked short streaks that are semi-transparent and hard to identify under light, and in severe cases, the whole leaf turns yellow or even reddish-brown. Dead heart refers to the occurrence during the booting or flowering stage of rice, caused by the borer boring the stem or internodes of rice. Since the images are captured by UAVs, they may contain interference areas such as weeds, soil, and other leaves, resulting in a complex recognition environment and further increasing the difficulty of identification.

### 2.2. Dataset Construction

**Figure 2:**
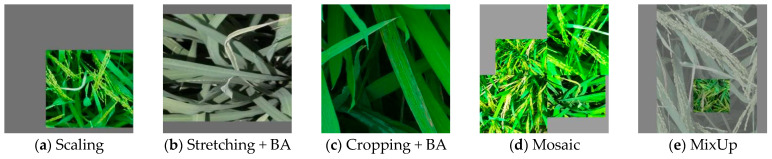
Partial sample data augmentation results.

The dataset was manually annotated using the LabelImg (1.8.6) annotation software, with labels for Leaf_roller (RLR), Leaf_blight (BLB), Bacterial_streak (BLS), and Dead_heart. Mosaic involved using four images, randomly cropping, scaling, rotating, etc., and synthesizing them into a single image, thereby augmenting the dataset while increasing the number of small samples. MixUp entailed rotating, scaling, etc., two images separately and stacking them to create new samples to balance the differences in sample quantities among different categories. To enhance the accuracy of the object detection algorithm, improve sample robustness, and boost model generalization capability, this paper applied random enhancements such as rotation, translation, scaling, shearing, brightness adjustment (BA), Mosaic, MixUp, etc., to the annotated original images [14,15], as shown in Figure 2 for some samples. After data augmentation, the dataset was expanded to 2000 images of diseases and pests. The dataset was divided into training, testing, and validation sets at an 8:1:1 ratio, completing the dataset construction.

## 3. Models and Training

### 3.1. YOLOv5 Algorithm

YOLOv5 is a deep learning model used for object detection, built upon the foundation of the YOLO (You Only Look Once) series of models. The backbone network of YOLOv5 utilizes a Concentrated-Comprehensive Convolution Block (C3) as the feature extraction module, which merges feature maps from different stages through cross-stage connections and partial connections to facilitate information transfer and exchange. Additionally, the C3 module employs channel shuffling by grouping channels to enhance interaction between features and enrich information content. Following the backbone network, YOLOv5 introduces the Path Aggregation Network (PANet) in the Neck network to fuse feature maps from different levels, thereby improving the performance of object detection across different scales. The overall architecture of YOLOv5 includes innovations and improvements in various aspects such as advanced backbone networks, feature fusion modules, and detection head design. This model has been widely applied in tasks like leaf-level disease and pest detection. However, there is still a need for further improvement in the performance of detecting diseases and pests at the canopy level, especially in complex backgrounds.

### 3.2. Improving YOLOv5 Algorithm

**Figure 3:**
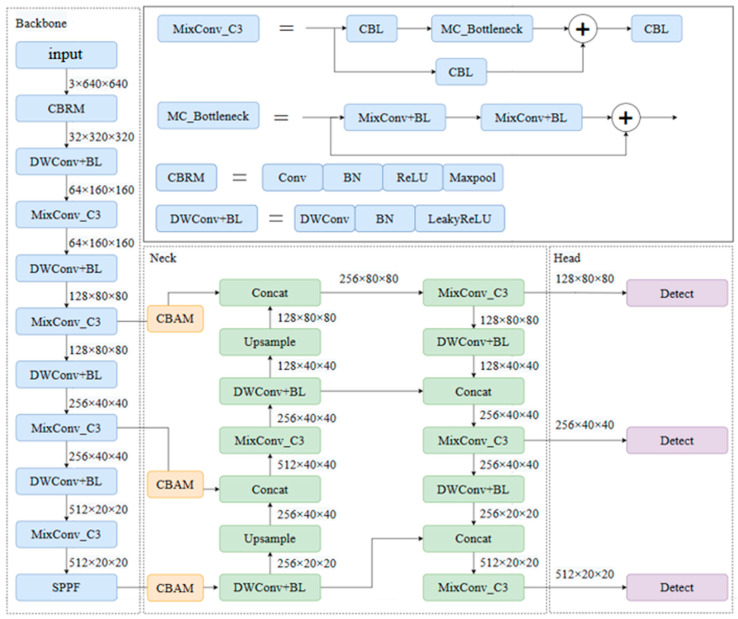
Improved YOLOv5 network model.

This paper intends to make improvements to the original YOLOv5 algorithm in five aspects, and the improved network structure is shown in Figure 3. The improvements are as follows:

1. Replace the first layer in the original YOLOv5 backbone network with CBRM. CBR includes normal convolution (Conv), Batch Normalization (BN), and the activation function (ReLU), where M represents the MaxPooling operation to reduce the feature map dimension. The MaxPooling operation achieves feature dimensionality reduction and position shift invariance by taking the maximum value within a window. Replace the normal convolution module in C3 with MixConv, which automatically selects the convolution kernel size most suitable for different feature sizes to enhance feature extraction ability and improve algorithm performance. Replace the downsampling module with separable convolution. Compared to traditional convolution operations, depthwise separable convolution consists of depthwise convolution and pointwise convolution, greatly reducing the model’s parameter and computation complexity.
2. Add the Convolutional Block Attention Module (CBAM) to the results of the three feature layers’ outputs by the main feature extraction network. By combining the channel attention mechanism and spatial attention mechanism, the CBAM can adaptively weight the feature maps, enhance effective feature information extraction, compress useless feature information, and improve the quality of feature representation.
3. Update the bounding box information of YOLOv5 through the k-means clustering algorithm to enable the network to learn better detectors, avoiding blind learning of target sizes and positions during the training process and improving the model’s detection performance.
4. Replace the original YOLOv5 loss function with the ComputeLossOTA, which comprehensively considers the existence, position, and classification accuracy of targets and helps the model continuously adjust its parameters. It provides better robustness for targets of different sizes, shapes, and categories, enabling better adaptation to complex scenarios and different target categories.
5. Change the activation function to LeakyReLU (0.1), introducing a small slope in the negative part to pass a certain gradient during backpropagation, helping alleviate the problem of gradient vanishing, especially in deep networks.

#### 3.2.1. MixConv

**Figure 4:**
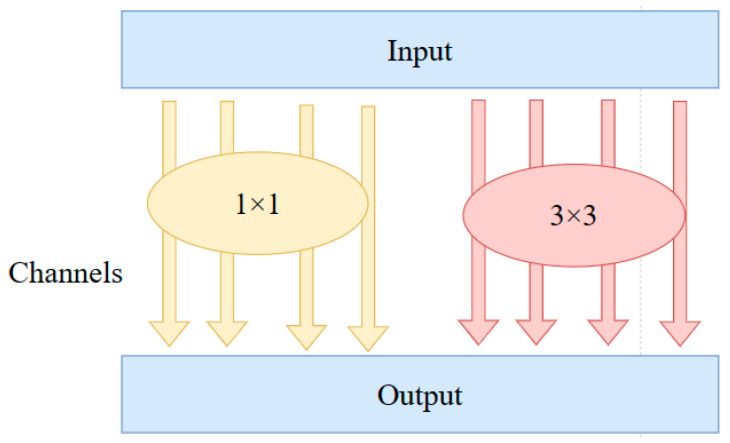
MixConv structural diagram. Note: MixConv divides channels into two groups, with convolution kernel sizes of 1 × 1 and 3 × 3, respectively.

The morphology, area, and size of lesions in the rice canopy leaves vary. The original YOLOv5 uses ordinary convolutions with fixed-size kernels to extract features, which cannot adapt to the extraction of fine features of lesions of different shapes and sizes. MixConv, on the other hand, enhances feature extraction by merging convolutions of different sizes [16], as shown in Figure 4. As depicted in Figure 4, MixConv first divides the input feature map into two sub-blocks based on the number of channels, then applies convolutions of different sizes to each sub-block, and finally concatenates the output feature maps to form a new mixed feature map. In this study, the convolution in the C3 module of the backbone network is replaced with mixed convolutions. By strengthening the model’s ability to extract different lesion feature information during feature extraction, the recognition accuracy of rice canopy diseases and pests is effectively improved.

#### 3.2.2. Depthwise Separable Convolutional

**Figure 5:**
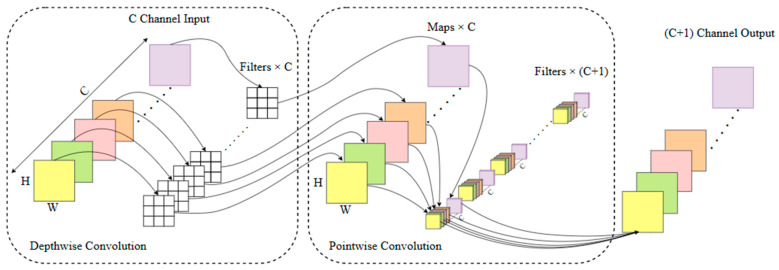
Depthwise separable convolutional structure diagram. Note: In the same feature map, different colors represent different features; In different feature maps, the same color represents the mapping result of the input features after convolution operation.

Depthwise separable convolution (DWConv) consists of two parts: depthwise convolution and pointwise convolution. In the depthwise convolution stage, the input feature map undergoes convolution with a 3 × 3 convolution kernel, but this convolution operation is performed separately on each input channel. In the pointwise convolution stage, the output feature map from the previous step is further processed. A 1 × 1 convolution kernel is used to weight and sum all channels at each pixel, resulting in a new output feature map. Figure 5 shows the structure diagram of depthwise separable convolution. Assuming the number of input and output feature channels is C and the convolution kernel size is 3 × 3, disregarding bias, the calculation of parameters for the depthwise separable convolution layer is 3 × 3 × C + 1 × 1 × C × C, while for a normal convolution layer it is 3 × 3 × C × C. The ratio of parameters between the former and the latter is 1/C + 1/9. Therefore, compared to traditional convolution operations, depthwise separable convolution reduces the model’s parameter and computation complexity, enhancing the model’s operational speed. By reducing the computational complexity of the model, depthwise separable convolution can run faster, making it particularly suitable for designing lightweight neural networks [17,18,19].

#### 3.2.3. CBAM

**Figure 6:**
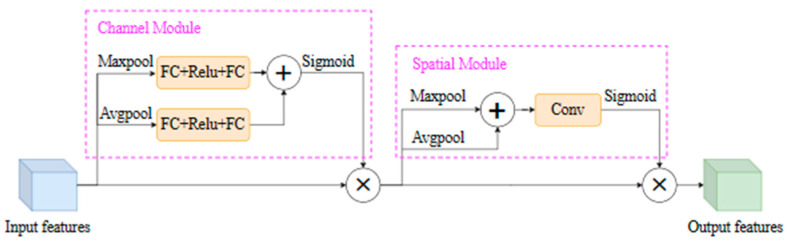
CBAM structural diagram.

The CBAM is an attention mechanism module consisting of a channel attention module and a spatial attention module. The input feature map is first processed through a channel attention module to obtain weighted results, and then it goes through a spatial attention module, ultimately producing weighted output, effectively enhancing the quality of feature representation. The principle of the channel module in Figure 6 is the same as that of the SE-Net module, consisting primarily of two parts: Squeeze and Excitation, with a focus on the channel attention mechanism [20]. Input features are separately passed through global max-pooling (Maxpool) and global average pooling (Avgpool) based on width and height, then through two fully connected layers and an activation layer (FC-ReLU-FC) for addition operation, followed by a sigmoid activation to obtain channel attention weights. These weights are then multiplied element-wise with the input features, ultimately generating the transitional features required for the Spatial Module. The Spatial Module part in Figure 6 refers to the spatial attention module. Taking the transitional features as the input features of this module, we take the sum of the results of Maxpool and Avgpool, separately, and then conduct a convolution operation to create one feature map. Finally, after passing through a sigmoid activation function, spatial attention weights are obtained. These spatial attention weights are multiplied with the transitional features, resulting in the generation of the combined channel and spatial attention mechanism features. The CBAM performs adaptive weighting on the input features in both the spatial and channel domains to enhance the quality of feature representation [21].

#### 3.2.4. K-Means Clustering Algorithm Updates Bounding Boxes

Bounding boxes are obtained from the ground truth boxes of the targets in the dataset, and they are used to guide the network in learning how to predict the position and category of targets. Bounding boxes have a significant impact on the accuracy of object detection networks, with higher detection accuracy achieved when the bounding boxes closely match the ground truth boxes. This paper uses the K-means clustering algorithm to extract bounding boxes suitable for the dataset. K-means is an unsupervised learning algorithm used to divide data points into K different clusters to identify the clustering structure of the data, where K represents the number of bounding boxes used and is set to 9.

The K-means clustering algorithm is used to cluster the ground truth boxes in the rice disease and pest dataset to determine the size and aspect ratio of the bounding boxes. Clustering is typically performed on the width and height of the ground truth boxes to find bounding boxes of different sizes and aspect ratios. The genetic algorithm is applied to mutate the results of the K-means clustering. The steps are as follows: (1) Initialize cluster centers by selecting 9 random ground truth boxes as the initial centers. (2) Calculate the distance between the ground true boxes of all the targets in the dataset and each cluster center. Assign the ground true boxes to the closest cluster center based on the distance and update the cluster center as the average of all the ground true boxes assigned to it. (3) Repeat the above steps, find the 9 optimal cluster centers as bounding boxes after 300 iterations, then randomly mutate the bounding boxes using the genetic algorithm, and optimize the bounding boxes based on a set of results with better performance.

**Figure 7:**
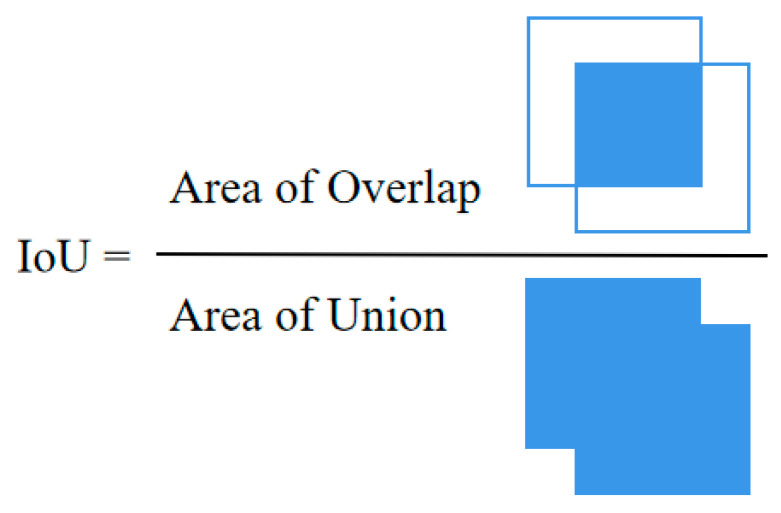
Calculation method for IoU.

The distance formula d = 1 − IoU, typically ranging from 0 to 1, where d = 0 indicates a very similar overlap between the ground truth box and the bounding boxes. Conversely, when there is no overlap between two bounding boxes, the distance d = 1, indicating they are dissimilar. The Intersection of Union (IoU) is as shown in Figure 7.

**Figure 8:**
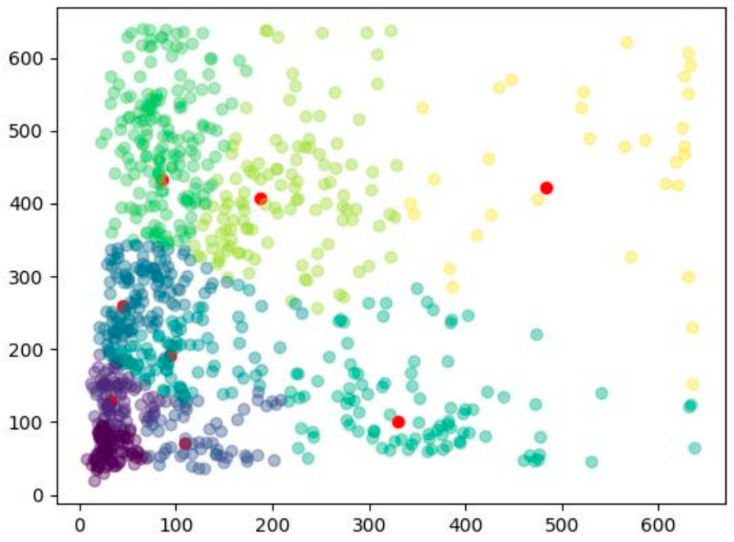
K-means clustering results of the ground truth box. Note: The 9 red dots represent the 9 cluster centers, and the canvas size is 640 × 640 pixels. Clusters of different colors represent the ground truth box of different features.

After clustering, 9 sets of bounding boxes are obtained, with a high fitness of 78% between the ground truth box and the bounding boxes. The K-means clustering result is shown in Figure 8.

#### 3.2.5. ComputeLossOTA

The original YOLOv5 loss function is the CIOU, which is mainly composed of the IoU, center point distance, and aspect ratio. In tasks where there are matching issues between multiple predicted boxes and ground truth boxes, the CIOU matches based only on IoU values, making it prone to situations where multiple predicted boxes simultaneously match a single ground truth box, leading to errors in target localization and classification. On the other hand, the ComputeLossOTA solves the matching problem using the optimal transport algorithm, ensuring that each ground truth target is matched with a predicted target, thereby avoiding such scenarios. When there are significant changes in target shape and size, the CIOU only considers the overlap between target boxes, making it susceptible to matching errors. In contrast, the ComputeLossOTA can better handle changes in target shape and size, improving the model’s robustness. Moreover, the ComputeLossOTA can also handle abnormal situations like partial occlusion or incomplete targets more effectively.

#### 3.2.6. LeakyReLU

**Figure 9:**
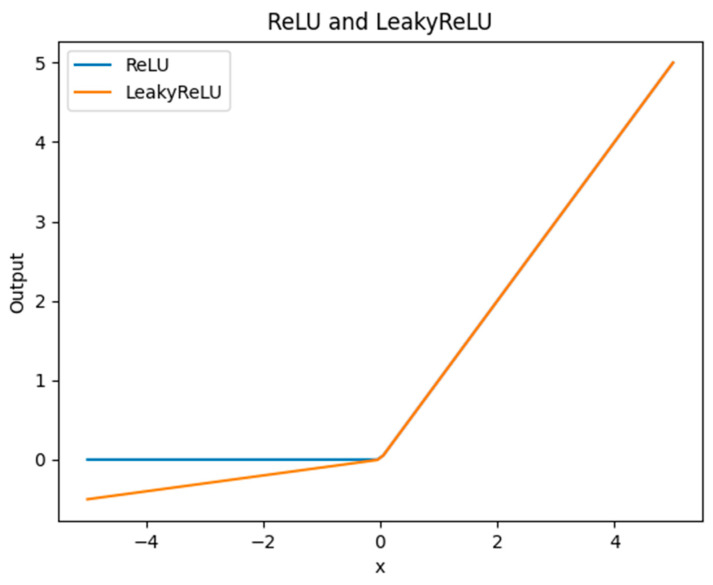
Comparison of ReLU and LeakyReLU activation functions.

The original YOLOv5 uses the ReLU activation function, which effectively solves the gradient vanishing problem. The ReLU has a gradient value of either 0 or 1. By truncating negative values to 0, ReLU introduces sparsity to the network, improving computational efficiency. However, ReLU outputs a constant 0 for negative inputs, which can lead to the issue of “dead” neurons. LeakyReLU (0.1) is a rectified linear unit with a negative slope. The parameter 0.1 represents the negative slope, allowing it to produce a small output for negative inputs. This helps alleviate the gradient vanishing problem while also reducing the problem of “dead” neurons. Figure 9 shows a comparison between the ReLU and LeakyReLU (0.1) activation functions. When the input value is greater than or equal to 0, the output results of ReLU and LeakyReLU (0.1) activation functions are the same. When the input value is less than 0, ReLU outputs constantly 0, while LeakyReLU (0.1) has a small slope, producing a non-zero gradient output to prevent the occurrence of “dead” neurons.

### 3.3. Model Training and Evaluation Metrics

#### 3.3.1. Training Platform

The main parameters of the training platform used in this experiment are as follows: Core i5-12400 CPU @ 4.4 GHz, Intel Corporation, Santa Clara, CA, USA, 32 GB RAM, 1 TB solid-state drive, Nvidia GeForce RTX 3070 24 GB graphics card, NVIDIA Corporation, Santa Clara, CA, USA, with CUDA and cuDNN versions 11.3 and 8.3.0 installed, running on the Windows 10 operating system. The Python version is 3.8, and the deep learning framework used is PyTorch 1.12.1.

#### 3.3.2. Training Parameters

Training Parameter Settings: the default image input size is set to 640 × 640, the batch size is 8, single-threaded, the initial learning rate is 0.01, and the training will run for 300 epochs.

#### 3.3.3. Evaluation Metrics

Detection models for rice diseases and pests have various evaluation metrics [22,23]. This paper adopts precision (*P*), recall (*R*), mean average precision (*mAP*), and frames per second (*FPS*) as evaluation metrics for the model. *P* is the ratio of true positive samples to the predicted positive samples, *R* is the ratio of *TP* samples to the actual positive samples, and *mAP* indicates the overall accuracy of the model identification. The formulas for *P* and *R* are shown in Equations (1) and (2).

(1) $$P=\sum_{i}^{N}\frac{{TP}_{i}}{{TP}_{i}+{FP}_{i}}\times 100\%$$

(2) $$R=\sum_{i}^{N}\frac{{TP}_{i}}{{TP}_{i}+{FN}_{i}}\times 100\%$$

where *N* represents the number of disease and pest categories for rice, which is 4. *TP_i_* (True Positives) refers to the number of positive samples correctly classified by the classifier for the *i*-th disease and pest category. *FP_i_* (False Positives) refers to the number of negative samples incorrectly classified by the classifier for the *i*-th disease and pest category. *FN_i_* (False Negatives) refers to the number of samples of the *i*-th disease and pest category that were not detected.

## 4. Result and Analysis

To compare and analyze the performance of the YOLOv5 and YOLOv5_DWMix models, three sets of experiments were designed under the condition of consistent model training parameters: a mainstream model performance comparison experiment, an attention mechanism comparison experiment, and an ablation experiment. Representative image data were selected for testing, and the detection results of the models were analyzed through visualization to evaluate the model’s performance.

### 4.1. Comparison of Classic Model Performance

YOLO is a typical one-stage model in the field of object detection. Compared to traditional object detection algorithms, it adopts a new approach by transforming the object detection task into a regression problem. It uses a single neural network to predict the entire image, rather than processing in stages as traditional algorithms do. This approach enables YOLO to achieve real-time detection while maintaining high detection accuracy, giving it an advantage in scenarios requiring high-speed processing [24,25]. On the other hand, Faster R-CNN is a typical representative of two-stage object detection models. It is a region-based object detection algorithm that generates a large number of image regions likely to contain objects using the region proposal method, and then it performs classification and localization based on these regions. The method is mainly divided into two parts. First, the Region Proposal Network (RPN) analyzes the input image to obtain candidate regions, and then the classifier is used for object detection within these candidate regions [26,27]. In summary, this paper selects two typical models, YOLO and Faster R-CNN, for detecting rice diseases and pests, and conducts a comparative analysis of the identification results. The selected models include YOLOv5, YOLOv5_MobileNet, YOLOv5_Ghost, YOLOv7, Faster_RCNN1 (vgg16), and Faster_RCNN2 (resnet50), totaling six models, and the recognition results are shown in Table 1.

The comparative experimental results show that *P*, *R*, and other indicators of the four YOLOv5 series models are higher than those of the Faster_RCNN model, with the YOLOv7 model performing the lowest. The YOLOv5_DWMix model performs the best in terms of *P*, *R*, and *mAP*, reaching levels of 95.8%, 95.1%, and 98.7%, respectively. At the same time, its *FPS* is relatively high, processing 25.93 frames per second. Compared to the YOLOv5 model, the *P*, *R*, and *mAP* have increased by 4.8%, 4.2%, and 3.4%, respectively, and the *FPS* has more than doubled. Compared to the original YOLOv5, YOLOv5_MobileNet and YOLOv5_Ghost use MobileNet and Ghost lightweight modules in the backbone network, respectively, to facilitate porting the trained model to mobile devices. Therefore, although there is a slight decrease in P, there is a significant improvement in *FPS*. Compared to YOLOv5, YOLOv7 has made significant improvements in network architecture. The feature extraction part utilizes the Multi_Concat_Block module, which includes two additional convolutional layers compared to the C3 module in YOLOv5. And there are also substantial improvements in the feature dimension reduction part. The YOLOv7 model has a more complex structure, requires more storage space, has higher GPU requirements, and performs poorly in all aspects of the rice disease and pest dataset. The Visual Geometry Group (VGG) structure is simple, consisting of multiple convolutional and pooling layers, but it lacks skip connections, which may lead to gradient vanishing issues. The Residual Network (ResNet50) introduces residual modules, effectively addressing gradient vanishing and model degradation problems in deep networks. However, it does not consider differences in target sizes and feature information at different scales. Therefore, the Faster_RCNN model with VGG and ResNet50 as backbone networks did not achieve good recognition results.

### 4.2. Performance Comparison of Different Attention Mechanisms

Introducing attention mechanisms can bring various benefits in object detection, including improving localization accuracy, enhancing focus on important targets, improving multi-scale detection, enhancing the ability to handle complex backgrounds, improving object classification capabilities, and increasing detection efficiency. By properly designing and applying attention mechanisms, the performance and robustness of object detection models can be further improved [28,29,30,31]. To verify the effectiveness of the CBAM, it is compared with four classic attention mechanism modules, namely, the Similarity Attention Module (SimAM), SE, channel attention (CA), and Efficient Channel Attention (ECA), as shown in Table 2.

**Figure 10:**
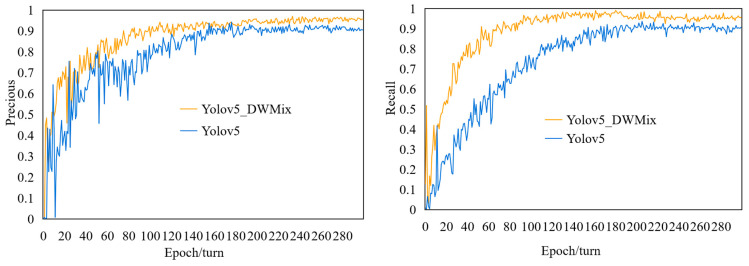
Comparison chart of accuracy and recall curves between YOLOv5_DWMix and YOLOv5 models.

From Table 2, it can be seen that the CBAM used in the improved YOLOv5_DWMix model outperforms the other four attention mechanisms. The CBAM performs the best in terms of *P*, *R*, and *mAP*, reaching 95.8%, 95.1%, and 98.7%, respectively. Compared to the SimAM, SE, CA, and ECA models, the accuracy of YOLOv5_DWMix has improved by 1.1%, 1.5%, 2.7%, and 0.2%, respectively. This indicates that the CBAM is better at selecting effective feature information, and its ability to identify rice diseases and pests is higher than that of SimAM, SE, CA, and ECA, effectively improving the model’s recognition accuracy and validating the feasibility of this approach. The *P* and *R* comparison curves obtained by iterating the improved algorithm with the original YOLOv5 for 300 epochs are shown in Figure 10. It is evident from Figure 10 that YOLOv5_DWMix achieves better *P* and *R* compared to the original YOLOv5.

### 4.3. Comparison of Ablation Experiments Performance

This paper mainly makes improvements in the following five aspects:

1. Regarding data preprocessing, image augmentation is applied to generate more diverse training samples, thereby expanding the dataset, reducing overfitting issues, and enhancing the model’s generalization ability.
2. In the backbone network, the first layer utilizes the CBRM module, which achieves dimension reduction of features and translation invariance of positions through max pooling operations. The MixConv is used to replace the regular convolutions in the C3 module. The DWConv is employed for feature downsampling to enhance feature extraction capabilities and improve algorithm performance.
3. The CBAM is added to adaptively weight the feature maps, enhancing the extraction of effective feature information while compressing irrelevant feature information to improve the quality of feature representation.
4. The bounding box information in YOLOv5 is updated using the k-means clustering algorithm.
5. The ComputeLossOTA function is utilized as the loss function, considering the key factors of object existence, position, and classification accuracy. LeakyReLU (0.1) serves as the activation function, effectively alleviating the problem of gradient disappearance.

To verify the effectiveness of the above improvements in the recognition of rice diseases and pests, successive ablation experiments are conducted with *P* as the evaluation metric, and the results are shown in Table 3.

Through the ablation tests, it is evident that, compared to Test 1, introducing image augmentation operations in Test 2 resulted in an increase of 0.9 percentage points in precision. By applying various image augmentation techniques, such as scaling, brightness adjustment, MixUp, Mosaic, etc., more diverse training samples can be generated. This not only expands the dataset but also effectively mitigates overfitting issues and enhances the model’s generalization ability. Compared to Test 2, after improving the backbone network in Test 3, precision increased by another 1.1 percentage points. The backbone network is the core part of the object detection model responsible for extracting image features. The improved backbone network enhances the model’s ability to represent targets, greatly boosting model performance. In comparison to Test 2, introducing the CBAM in Test 4 further increased precision by 0.7 percentage points. The CBAM enhances feature representation through channel and spatial attention mechanisms, effectively strengthening the model’s perception and discrimination capabilities toward targets. Compared to Test 4, updating the bounding boxes in Test 5 allowed the model to better adapt to rice disease and pest lesions of different scales and shapes, resulting in a 0.4 percentage point increase in precision. Test 6, compared to Test 5, introduced the ComputeLossOTA and LeakyReLU (0.1) functions, leading to a 0.5 percentage point increase in precision. Overall, compared to the original YOLOv5, the improved model YOLOv5_DWMix has achieved a total increase in *P* of 4.8 percentage points. Ablation experiments demonstrate the impact of each component on the performance of the object detection model. The gradual introduction of different components has consistently improved the model’s precision, highlighting the importance of these components in object detection tasks.

### 4.4. Identification Results of Rice Diseases and Pests Using Different Models

**Figure 11:**
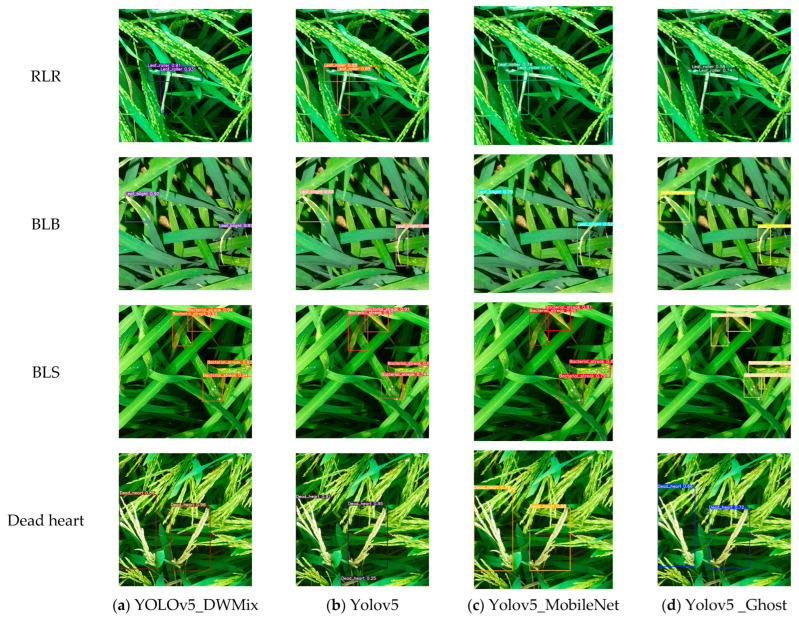
Comparison of recognition results of different models.

After comparing several classic models, including YOLOv5_DWMix, YOLOv5, YOLOv5_MobileNet, and YOLOv5_Ghost, it was found that these four models performed well. These four trained models were used to detect rice diseases and pests in natural scenes, and some of the results are shown in Figure 11. From Figure 10, the overall average accuracy of the four models follows the order YOLOv5_DWMix > YOLOv5 > YOLOv5_MobileNet > YOLOv5_Ghost. The YOLOv5_DWMix model generally exhibits higher recognition accuracy for all four types of diseases and pests, while the YOLOv5 and YOLOv5_MobileNet models show better recognition accuracy for dead heart compared to the other three types. Based on the BLS recognition results, it is evident that the YOLOv5_DWMix model performs well in detecting smaller lesions, whereas the other three models show poorer performance in detecting small lesions. In summary, the YOLOv5_DWMix model demonstrates the best performance in identifying rice diseases and pests.

## 5. Discussion

With the rapid proliferation of UAV technology and the continuous advancement of deep learning (DL) algorithms, the integration of high-resolution images acquired by UAVs with DL methods offers a powerful approach to monitoring rice diseases and pests. This technology enables large-scale, comprehensive monitoring of crop growth and the distribution of diseases and pests, providing significant benefits for precision agriculture. However, to successfully implement this model in real-world rice fields, several critical factors need to be addressed [32,33,34]. First, the model must be integrated into the UAV’s onboard computing system, which may require interfacing with existing software platforms like Pix4D or Agisoft PhotoScan Pro. This integration is essential for enabling seamless data collection and processing. Second, the memory and computational demands of the model will vary depending on its complexity and the type of hardware used. Optimizing the model’s size is crucial to ensure real-time inference capabilities on the UAV. Additionally, converting the model into a more compact and efficient format, such as ONNX (Open Neural Network Exchange), can further enhance its performance and applicability. Beyond hardware and software considerations, practical challenges such as power consumption, real-time processing capabilities, and connectivity need to be carefully addressed. UAVs are typically constrained by battery life, making computational efficiency a critical factor for maintaining performance during extended flight periods.

Looking ahead, future research should focus on the integration of multi-source and multi-modal data fusion techniques to further improve the model’s generalization ability and robustness. Given the complex and variable nature of field environments and regional differences, incorporating additional data sources, such as geographic and meteorological information, could significantly enhance the accuracy and applicability of monitoring systems across diverse scenarios. Moreover, combining multiple data modalities, such as visible light, multispectral, and hyperspectral data from various sensors, has the potential to further boost the efficiency and accuracy of rice disease and pest monitoring [35,36]. By addressing these challenges and exploring these future research directions, the deployment of UAV-based monitoring systems can be significantly improved, leading to more effective and efficient crop management practices.

## 6. Conclusions

The YOLOv5_DWMix model demonstrated significant improvements in the detection speed and precision of rice canopy diseases and pests. Experiments revealed that the model effectively identified the diseases and pests with high accuracy.

To improve model performance, the training dataset was enhanced through image augmentation techniques, generating a more diverse dataset. The anchor box information of the YOLOv5 model was updated using the k-means clustering algorithm to better align with the distribution of objects in the dataset. To enhance feature extraction, the CBAM was introduced, enabling the model to focus on the most relevant features. The ComputeLossOTA loss function was employed to ensure more accurate bounding boxes by improving the model’s precision. LeakyReLU (0.1) was used as the activation function to address the issue of dead neurons. The YOLOv5_DWMix model was compared to several classic models. The results demonstrated substantial improvements in *P*, *R*, *mAP*, and *FPS*. The YOLOv5_DWMix model consistently outperformed the other models in all evaluation metrics, confirming its effectiveness in identifying rice canopy diseases and pests.

## Figures and Tables

**Table 1:** Training results of classical model.

Model	*P*/%	*R*/%	*mAP*/%	*FPS*/s
YOLOv5_DWMix	95.8	95.1	98.7	25.93
YOLOv5	91	90.9	95.3	11.78
YOLOv5_MobileNet	86.7	91.8	93.0	17.68
YOLOv5_Ghost	81.8	88.9	90.2	18.7
YOLOv7	62.6	51.0	69.6	10
Faster_RCNN1 (vgg16)	71.2	48.5	76.2	9.46
Faster_RCNN2 (resnet50)	70.4	47.9	74.1	7.57

**Table 2:** Training results of different attention mechanism models.

Model	*P*/%	*R*/%	*mAP*/%
CBAM	95.8	95.1	98.7
SimAM	94.7	94.8	98.4
SE	94.3	97.4	98.9
CA	93.1	94.7	96.5
ECA	95.6	96.3	98.3

**Table 3:** Experimental results of different ablation methods.

Test	Image Augmentation	Backbone Network	CBAM	K-Means	Loss + Activation Function	*P*/%
1	-	-	-	-	-	92
2	√	-	-	-	-	92.9
3	√	√	-	-	-	94.0
4	√	√	√	-	-	94.7
5	√	√	√	√	-	95.1
6	√	√	√	√	√	95.6

## Data Availability

The author is currently conducting research on unmanned aerial vehicle remote sensing recognition of crop diseases and pests, and the dataset mentioned in the article is a part of it, so the dataset is limited. To access the dataset, please contact the author.
